# Severe Abdominal Injuries without External Wound

**Published:** 1898-10-29

**Authors:** 


### SEVERE ABDOMINAL INJURIES WITHOUT EXTERNAL WOUND.

At the meeting of the Medical Society of London held last Monday, Mr. Marmaduke Sheild read a paper in which he described four cases in which he had performed abdominal section for severe abdominal injuries which had not been associated with external wound. In one a rent was found in the mesentery, in a second the jejunum was torn across, in a third there was laceration of the upper surface of the liver, and in a fourth the injury was not discovered at the time of the operation. Except the first case all died; nevertheless the author maintained that this should not deter one from operation in suitable cases, the indications being the persistence of collapse, with the occurrence of distension and rigidity of the abdominal walls. The subject is an all important one, the high mortality of severe contusions of the abdomen being proverbial. Recent American teaching, an account of which want of space compels us to postpone till next week, fully supports Mr. Sheild, and seems even to go still further.

